# P-1226. Growth Suppression Dynamics of Minocycline, Eravacycline, and Omadacycline Against Stenotrophomonas maltophilia Clinical Isolates

**DOI:** 10.1093/ofid/ofaf695.1418

**Published:** 2026-01-11

**Authors:** Ashlan J Kunz Coyne, Alex Do, Rachel Gray

**Affiliations:** University of Kentucky College of Pharmacy, Lexington, KY; University of Kentucky College of Pharmacy, Lexington, KY; University of Kentucky College of Pharmacy, Lexington, KY

## Abstract

**Background:**

*Stenotrophomonas maltophilia* is an opportunistic pathogen with limited treatment options. Minocycline (MIN) is considered a first-line therapy. Omadacycline (OMC) and eravacycline (ERV) also show in vitro activity, but comparative dynamic data are limited.

**Figure:**
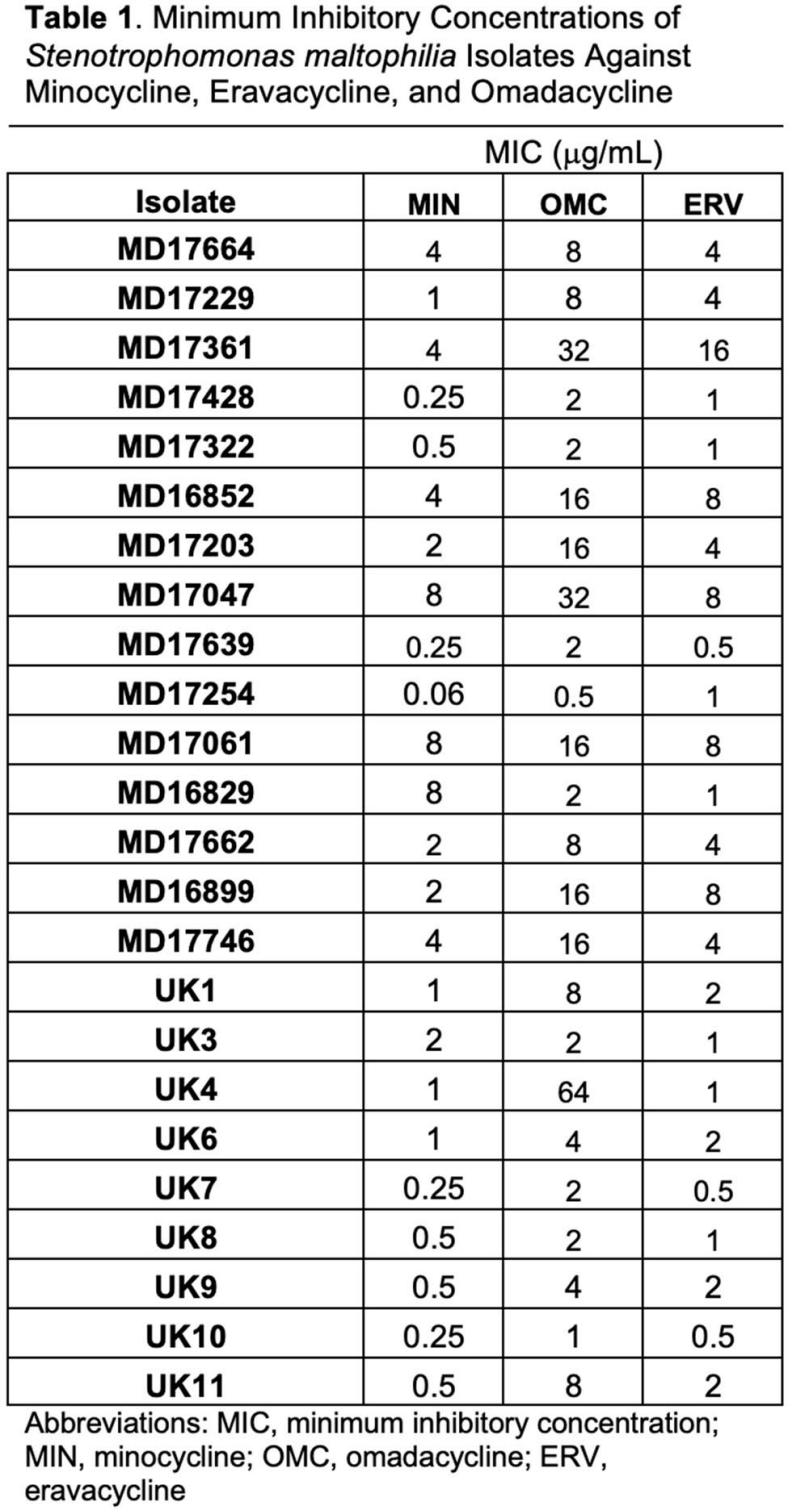

**Figure:**
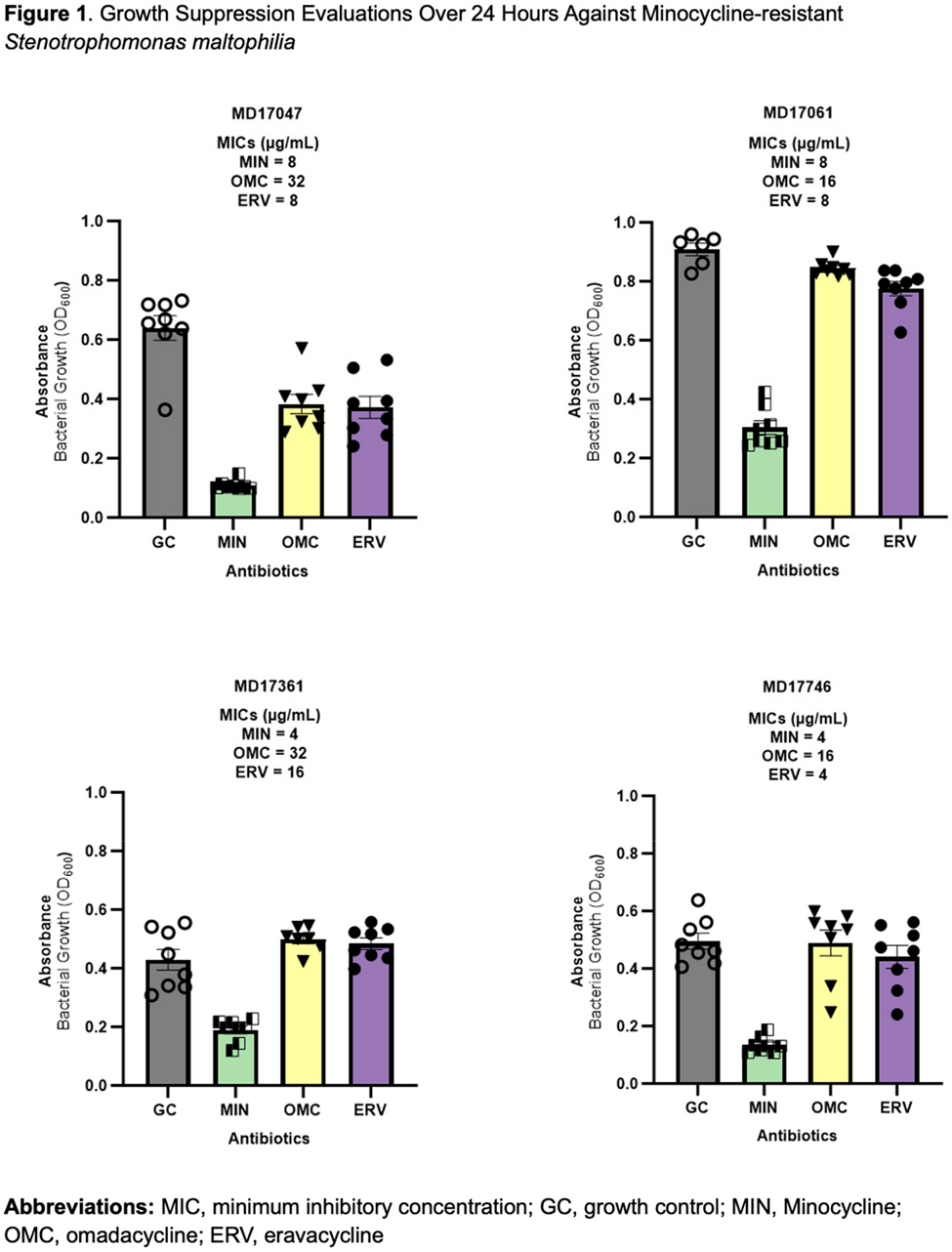

**Methods:**

Twenty-five whole-genome sequenced *S. maltophilia* isolates (15 from hematologic malignancy patients at MD Anderson, 10 from chronic respiratory disease patients at UK HealthCare) underwent MIC testing via broth microdilution per CLSI guidelines against MIN, OMC, and ERV. MICs were tested in triplicate and interpreted using CLSI breakpoints where available. Dynamic growth inhibition was assessed over 24 hours using OD_600_ readings every 15 minutes in a Cytation7 system with shaking at 37°C. Cmax concentrations were used with an inoculum of 10⁷ CFU/mL.

**Figure:**
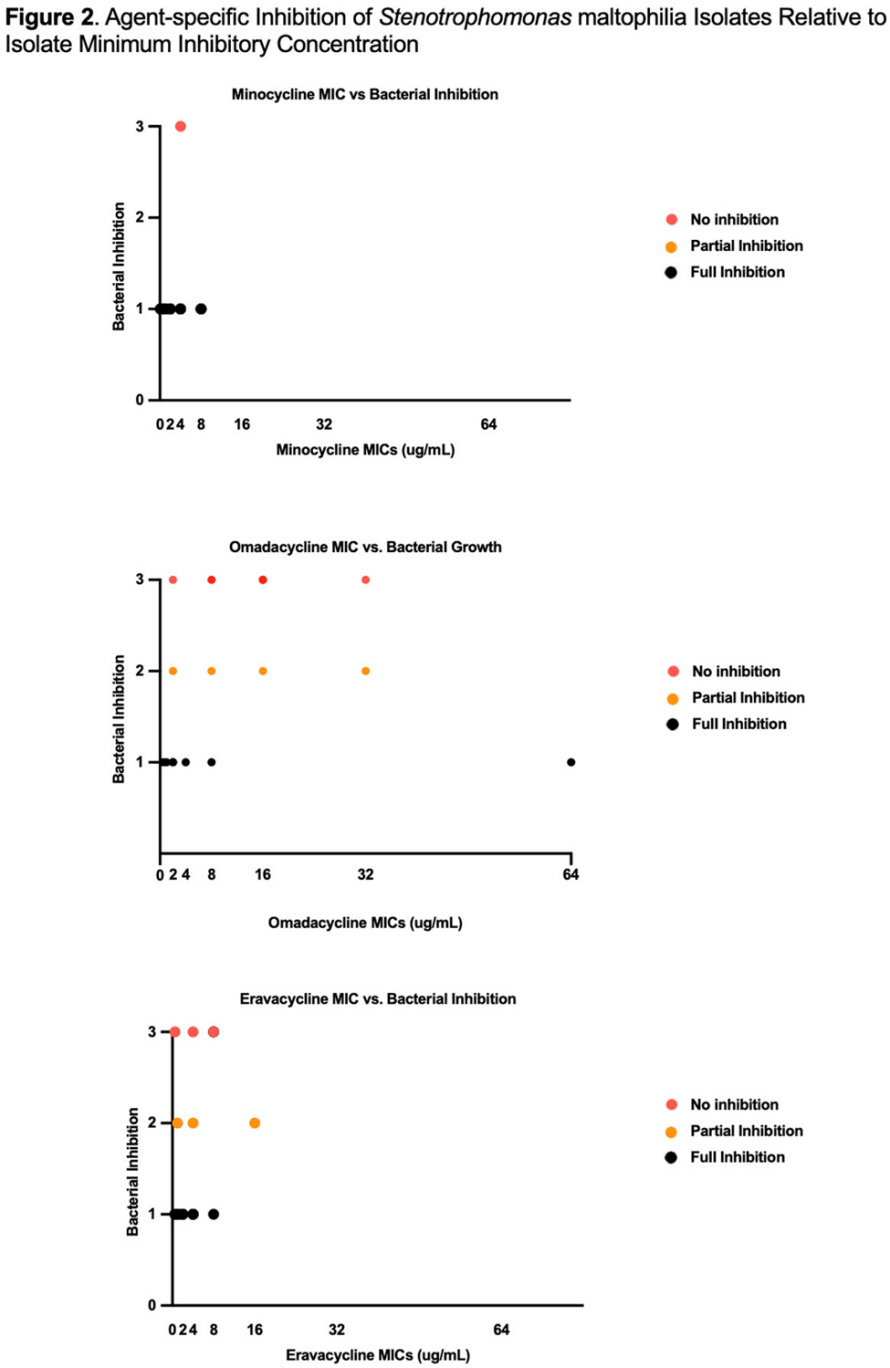

**Results:**

MIC_50_/MIC_90_ values were 1/8 µg/mL for MIN, 8/32 µg/mL for OMC, and 2/8 µg/mL for ERV. In 24-hour growth assays, MIN consistently reduced growth, including in isolates with MICs of 4–8 µg/mL. ERV produced partial suppression across all tested isolates but was less potent. OMC showed minimal inhibition, often comparable to growth controls. In Figure 2, MIN showed full inhibition in most isolates, even at elevated MICs. OMC activity declined sharply with increasing MICs, with no inhibition at MICs ≥8 µg/mL. ERV maintained partial or full inhibition over a wider range, though full inhibition was less frequent. Notably, ERV retained activity even in isolates with MICs of 8–16 µg/mL, while OMC failed to suppress growth despite lower MICs in some cases. This suggests MIC alone may not reliably predict in vitro efficacy.

**Conclusion:**

MIN showed the most consistent dynamic inhibition. ERV offered moderate activity across MICs, while OMC lacked reliable suppression. These findings support continued use of MIN and suggest a potential role for ERV in *S. maltophilia* treatment.

**Disclosures:**

All Authors: No reported disclosures

